# A Novel Luciferase-Based Reporter Gene Technology for Simultaneous Optical and Radionuclide Imaging of Cells

**DOI:** 10.3390/ijms25158206

**Published:** 2024-07-27

**Authors:** Natasa Gaspar, Maryana Handula, Marcus C. M. Stroet, Kranthi Marella-Panth, Joost Haeck, Thomas A. Kirkland, Mary P. Hall, Lance P. Encell, Simone Dalm, Clemens Lowik, Yann Seimbille, Laura Mezzanotte

**Affiliations:** 1 Department of Radiology and Nuclear Medicine, Erasmus MC Cancer Institute, University Medical Center Rotterdam, 3015 CE Rotterdam, The Netherlands; 2 Department of Molecular Genetics, Erasmus MC Cancer Institute, University Medical Center Rotterdam, 3015 CE Rotterdam, The Netherlands; 3 Promega Corporation, Madison, WI 53711, USA

**Keywords:** bioluminescence imaging, luciferase complementation, radionuclide imaging, reporter gene, peptide tracers

## Abstract

Multimodality reporter gene imaging combines the sensitivity, resolution and translational potential of two or more signals. The approach has not been widely adopted by the animal imaging community, mainly because its utility in this area is unproven. We developed a new complementation-based reporter gene system where the large component of split NanoLuc luciferase (LgBiT) presented on the surface of cells (TM-LgBiT) interacts with a radiotracer consisting of the high-affinity complementary HiBiT peptide labeled with a radionuclide. Radiotracer uptake could be imaged in mice using SPECT/CT and bioluminescence within two hours of implanting reporter-gene-expressing cells. Imaging data were validated by ex vivo biodistribution studies. Following the demonstration of complementation between the TM-LgBiT protein and HiBiT radiotracer, we validated the use of the technology in the highly specific in vivo multimodal imaging of cells. These findings highlight the potential of this new approach to facilitate the advancement of cell and gene therapies from bench to clinic.

## 1. Introduction

Reporter gene imaging represents a straightforward approach for both tumor detection and the evaluation of cell-based therapies (e.g., stem cells or immune cells) in preclinical studies. Multimodality molecular imaging offers high sensitivity, resolution and translational potential by combining the benefits of optical, nuclear and magnetic resonance imaging techniques [1,2,3].

Following recent breakthroughs with their efficacy in clinical studies and their resulting approval, gene and cell-based therapies represent an exciting new area in treatment innovation. However, our ability to monitor the biodistribution of therapeutics, detect early toxicities and evaluate treatment efficacy in a simple, non-invasive manner will be critical for the continued development and, ultimately, the success rate of these new approaches [4].

Reporter gene sequences are amenable to incorporation into gene therapy vectors and the specific integration into safe harbors in the genomes of therapeutic cells. Moreover, unlike direct labeling methods, the signals from genetic reporters are not diluted with time and do not pose the potential for altering the metabolism of therapeutic T cells [4]. There are already several reporter genes commonly used for preclinical visualization of engineered cells that enable fluorescence (FLI) and bioluminescence (BLI) imaging [5,6,7]. These reporters are also used for translational modalities, such as positron emission tomography/single photon emission computed tomography (PET/SPECT), magnetic resonance imaging (MRI) and photoacoustic imaging [8,9,10]. The genetic reporters are usually co-expressed as fusion proteins or in tandem to achieve multimodality imaging [11]. The coupling of two imaging techniques with complementary attributes is attractive, as it can in principle compensate for limitations associated with the individual methods.

One such approach makes use of specific reporter genes for each imaging modality that can be detected in animals by subsequent injections of bioluminescence substrates or radiotracers [12,13]. The benefits of using BLI in preclinical studies rely on the specificity derived from high signal-to-noise ratio, short imaging times and cost (no need for dedicated personnel and contained facilities). In contrast to optical methods, such as bioluminescence, radionuclide imaging is not prone to signal attenuation in deeper tissues. It is therefore preferred for the imaging of deep tissue. Another important distinction of radionuclide imaging is its translational potential. When used either separately or together, both bioluminescence and radionuclide technologies enable repeated, non-invasive image acquisitions that can be collected from the same subject over time.

Nuclear reporter genes have been recently used in the clinic for tracking of therapeutic cells [14,15]. Specifically, cytotoxic T cells have been engineered to express both a chimeric antigen receptor (CAR) for targeting glioma cells as well as herpes simplex virus type 1 thymidine kinase (HSV1-TK) [14]. This dual reporter-suicide gene enables selective uptake of the PET tracer [^18^F]FHBG for tracking of localization and controlling the viability of administered cells in glioma patients. Other reporter genes were shown to have predictive value for cell-based treatments in preclinical models with solid tumors [16,17,18], e.g., the human hNIS reporter gene (2.2 Kb) has been shown to be a valid reporter for CAR T cell therapy monitoring in a preclinical study [17].

**Figure 1:**
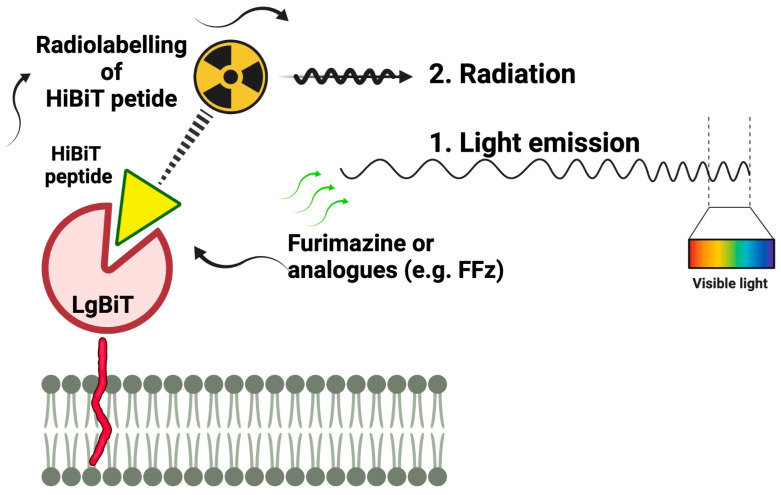
Schematic representation of the novel reporter gene technology. LgBiT protein is anchored to the membrane with PDGFR transmembrane domain and exposed to the extracellular space.

Herein, we developed a chimeric reporter gene system that enables simultaneous optical (BLI) and nuclear (SPECT/PET) gene and cell visualization. Our reporter gene encodes a reporter protein (LgBiT) that represents the large fragment of the bioluminescent split system derived from the NanoLuc luciferase [19]. NanoLuc is a small luciferase (19 kDA) that produces a 100-fold brighter signal than *Renilla* or firefly luciferase [20]. LgBiT is expressed and anchored on the membrane of engineered cells via fusion to the C-terminal transmembrane anchoring domain of platelet-derived growth factor receptor (PDGFR) (Figure 1). The small size of the reporter gene (800 bp) is amenable to the design of viral vectors appropriate for multimodal and highly specific imaging of cells. Because of high affinity between LgBiT and the complementary 11-amino acid (aa) HiBiT, the peptide can be labeled with radionuclides to target LgBiT on the cell membrane, making it attractive for SPECT imaging [19,21]. Tracer peptides used for this purpose are generally small (<50 aa), easy to synthesize, possess high target selectivity, display rapid pharmacokinetics and clearance and are non-immunogenic [22]. Clearance ought not to be too rapid to induce nephrotoxicity. The delicate balance between these attributes and potential liabilities is critical for their performance in vivo and in the clinic [22,23].

The purpose of this study was to evaluate the combined potential of TM-LgBiT, a chimeric reporter gene, and a novel tracer, DOTA-6-Ahx-HiBiT (VSGWRLFKKIS), for dual modality SPECT/BLI in vivo imaging (Figure 1). To explore this, we engineered a chimeric membrane reporter gene (TM-LgBiT) as well as a DOTA-6-Ahx-HiBiT (VSGWRLFKKIS) subsequently labeled with indium-111. The resulting reporter reagents enabled combined radionuclide (SPECT/PET) and bioluminescence (BLI) imaging in cells and small animals, demonstrating for the first time the bridging together of optical nuclear imaging modalities.

## 2. Results

### 2.1. Synthesis and Radiolabeling of DOTA-6-Ahx-VSGWRLFKKIS

For the tracer design we used a flexible linker to conjugate HiBit peptide, VSGWRLFKKIS, to the DOTA chelator. The preparation of the peptide sequence, VSGWRLFKKIS, was performed following a *Nα*-Fmoc solid-phase peptide synthesis strategy (Supplementary Figure S1). The coupling of the linker to the *N*-terminal valine residue was established using a commercially available Fmoc-6-Ahx-OH using standard coupling agent. The conjugation of the chelate was carried out using a commercially available DOTA-tris (*t*Bu) ester under basic conditions in the presence of PyBOP (Supplementary Figure S1). Cleavage of the peptide from the solid support and concomitant removal of the protecting groups was performed under acidic conditions. Purification of the crude compound resulted in 16.8% yield. Radiolabeling of DOTA-6-Ahx-VSGWRLFKKIS was set up with [^111^In]InCl_3_ to give 97% radiochemical yield and a molar activity corresponding to 150 MBq/nmol. Stability studies were performed by incubation of [^111^In]In-DOTA-6-Ahx-VSGWRLFKKIS in PBS and mouse serum for up to 4 h. The results showed that about 90% of the peptide remained intact up to 4 h after incubation in PBS, indicating that the compound is only mildly sensitive to radiolytic degradation over this amount of time. However, in mouse serum, only 71% of the peptide remained after 4 h, suggesting that it could be less stable in a live animal (Supplementary Table S1). The lipophilicity of [^111^In]In-DOTA-6-Ahx-VSGWRLFKKIS was determined by measurement of the LogD_7.4_ value. The obtained value, −2.03 ± 0.72, proves that our compound is hydrophilic and therefore more likely to be cleared by the kidneys (Supplementary Table S1).

### 2.2. Characterization of TM-LgBiT Reporter Gene and [^111^In]In-DOTA-6-Ahx-VSGWRLFKKIS (HiBiT) In Vitro

#### 2.2.1. Affinity

**Figure 2:**
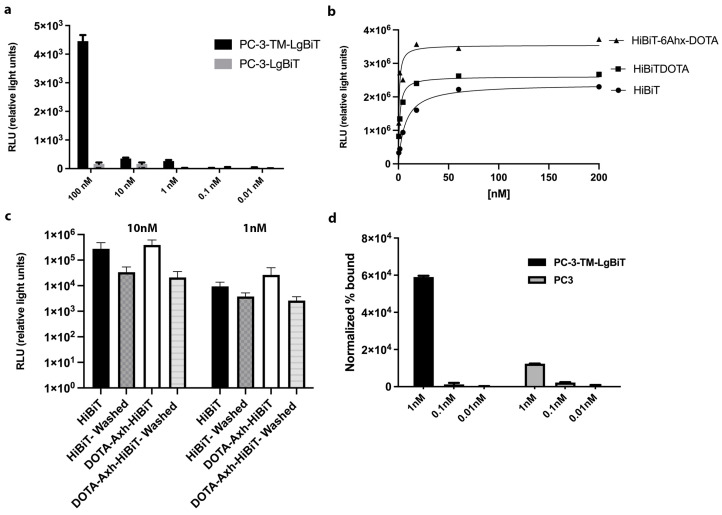
Characterization of TM-LgBiT reporter gene and HiBiT tracers. Luminescence signals were detected using the IVIS, validating the expression of reporter (LgBiT) when expressed on the membrane or within the cytosol of PC-3 cells upon administration of different concentrations of HiBiT peptide (**a**). To assess how the conjugation affects the HiBiT probe characteristics, functional and affinity data were obtained using one site specific binding function using purified LgBiT. Calculated K_D_ values were as follows: HiBiT = 6.8 nM; DOTA-VSGWRLFKKIS = 1.3 nM; and DOTA-6-Ahx-VSGWRLFKKIS = 0.7 nM. (**b**). DOTA-6-Ahx-VSGWRLFKKIS and HiBiT probes’ binding specificity was evaluated on tracer-positive (TM-LgBiT) and -negative PC-3 cells based on obtained luminescence signals. DOTA-6-Ahx-VSGWRLFKKIS and HiBit bioluminescence signal in TM-Lgbit PC-3 cells was not significantly different (**c**). Specific binding after 1 h of incubation with 1 nM/150 MBq of [^111^In]In-DOTA-6-Ahx-VSGWRLFKKIS in cells expressing TM-LgBiT in comparison to control cells. At 1 nM concentration, the radioactive signal of PC-3-TM-LgBiT cells is significantly higher than in PC-3 controls (*p*-value < 0.05). The results shown were performed in triplicate, and the values are indicated as means ± SD (**d**).

We first assessed the functionality of transmembrane TM-LgBiT protein when expressed anchored to the membrane via PDGFR transmembrane domain (TM-LgBiT) or within the cytosol of PC-3 cells using BLI imaging. Luminescence signals were detected using the IVIS, validating the expression of LgBiT in transduced PC-3 cells as well the complementation with HiBiT. We found that even the lowest HiBiT peptide concentration (10 pM) resulted in a 3-fold higher signal for the membrane anchored reporter vs. the intracellular one (Figure 2a). Next, the HiBiT peptide was linked to the DOTA chelator to enable radiolabeling for nuclear imaging purposes using different types of linkers. We evaluated the binding affinity of DOTA-VSGWRLFKKIS and DOTA-6-Ahx-VSGWRLFKKIS using one site specific binding function, as shown in Figure 2b. The calculated K_D_ values were 6.8 nM for intact HiBiT peptide (VSGWRLFKKIS), 1.3 nM for DOTA-VSGWRLFKKIS and 0.7 nM—showing the highest affinity for our conditions—for DOTA-6-Ahx-VSGWRLFKKIS (Figure 2b). All further studies were carried out with DOTA-6-Ahx-VSGWRLFKKIS due to its increased affinity toward TM-LgBiT compared to DOTA-VSGWRLFKKIS.

#### 2.2.2. Specificity

We further examined the binding specificity (using bioluminescence) of DOTA-6-Ahx-VSGWRLFKKIS and HiBiT on both control PC-3 and PC-3-TM-LgBiT cells (Figure 2c). After addition of the HiBiT peptide (10 nmol) to PC-3-TM-LgBiT cells (no wash), the signal was approximately one order of magnitude higher than it was in an assay performed in washed PC-3-TM-LgBiT cells (Figure 2c). Compared to the signals obtained with addition of the HiBiT peptide to the reaction, there were no significant differences in light output when incubating cells with DOTA-6-Ahx-VSGWRLFKKIS (10 nmol) or with PC-3-TM-LgBiT cells (Figure 2c). Observing the control cell line (PC-3), we did not notice any signal after addition of the HiBiT or DOTA-6-Ahx-VSGWRLFKKIS peptide. Signals obtained from PC-3 cells were at background levels under all conditions. When we performed a similar assay, by using radioactive [^111^In]In-DOTA-6-Ahx-VSGWRLFKKIS peptide, we obtained a significant 5-fold difference (*p*-value < 0.01) in radioactive signals between the expressing and non-expressing cells, after incubation and washing, by using a 1 nM concentration of peptide (Figure 2d).

### 2.3. Bioluminescence Imaging of TM-LgBiT-Expressing Cells

**Figure 3:**
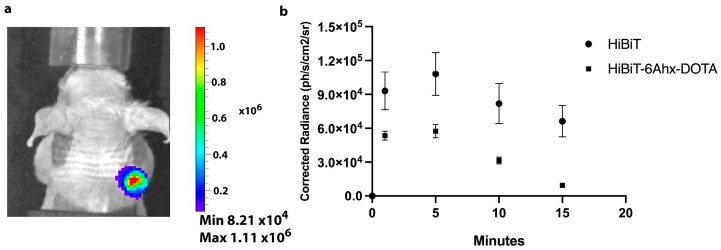
Longitudinal bioluminescence imaging (BLI) of HiBiT probe distribution in mice implanted with target (TM-LgBiT)-positive tumors (**a**). A strong and specific bioluminescent signal from NanoLuc luciferase was detected at the tumor site 30 min after peptide (HiBiT and DOTA-6-Ahx-VSGWRLFKKIS) injection upon administration of fluorofurimazine (minutes after fluorofurimazine injection indicated in the x axis) (**b**).

To assess reporter gene expression and the in vivo sensitivity with both the native HiBiT peptide and its synthetic analog DOTA-6-Ahx-VSGWRLFKKIS, we performed imaging studies using the IVIS in living mice (n = 6) by subcutaneously injecting control PC-3 cells (n = 3) and PC-3-TM-LgBiT (n = 3), stably expressing LgBiT reporter gene. The tumors were allowed to grow until they reached a palpable size of approximately ~4–6 mm in diameter. Prior to testing the in vivo system performance, we assessed the background emission of LgBiT expressed from tumor cells (PC-3-TM-LgBiT) upon intraperitoneal administration of NanoLuc substrate fluorofurimazne (1.3 µmol), allowing us to estimate the background signal of LgBiT protein/fluorofurimazine. An average background signal of 2.63 × 10^4^ ph/s was detected, mostly coming from the abdomen. After separate i.v. injections of HiBiT peptides (native and DOTA-6-Ahx-VSGWRLFKKIS), the NanoBiT complex was reconstituted successfully; it formed a functional enzyme, resulting in specific light emission coming from the PC-3-TM-LgBiT tumors (Figure 3a). Moreover, we detected a strong and specific bioluminescent signal emerging from NanoLuc luciferase reconstitution (10-fold higher than previously detected background) at the tumor site 30 min after injection of the peptide (DOTA-6-Ahx-VSGWRLFKKIS or VSGWRLFKKIS) (Figure 3b). These data were consistent across all mice bearing PC-3-TM-LgBiT tumors, showing that we could detect reporter gene expression and complementation with exogenously administered peptide in vivo via BLI imaging.

### 2.4. In Vivo Combined BLI and SPECT/CT Scan and Biodistribution

**Figure 4:**
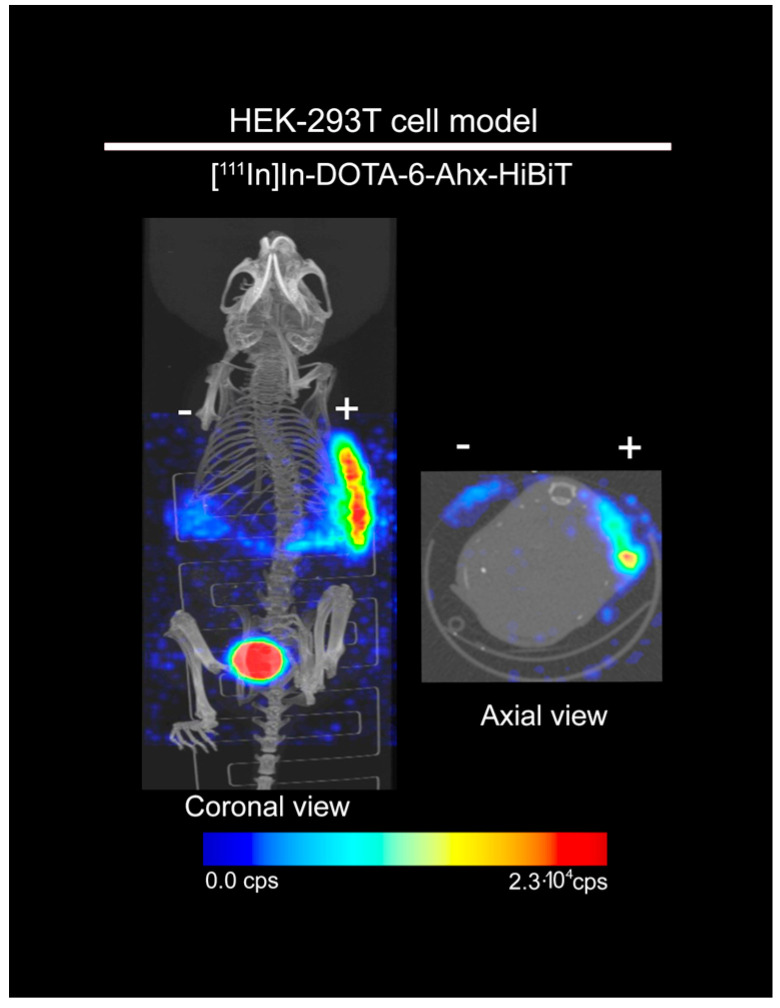
[^111^In]In-DOTA-6-Ahx-VSGWRLFKKIS SPECT/CT imaging of mice implanted with target-positive and -negative HEK-293T cells. [^111^In]In-DOTA-6-Ahx-VSGWRLFKKIS showed higher uptake (3.6-fold) in HEK-293T target-positive cells (right flank, +) compared with the target-negative HEK-293T cells (left flank, −) at 1 h after subcutaneous administration of [^111^In]In-DOTA-6-Ahx-VSGWRLFKKIS probe.

In order to test radionuclide imaging using SPECT/CT, we performed the initial experiments using HEK-293T-TM-LgBiT cells. BALB/c nude mice were injected with HEK-293T cells expressing TM-LgBiT (right flank) and HEK-293T control cells (left flank) (n = 3). The mice were subjected to a 1 h dynamic SPECT/CT after subcutaneous administration of 0.13 nmol/20 MBq of [^111^In]In-DOTA-6-Ahx-VSGWRLFKKIS. HEK-293T-LgBiT cells showed specific uptake of the radiotracer with approximately 3.6-fold higher uptake than control HEK-293T at 1 h after tracer injection (Figure 4).

**Figure 5:**
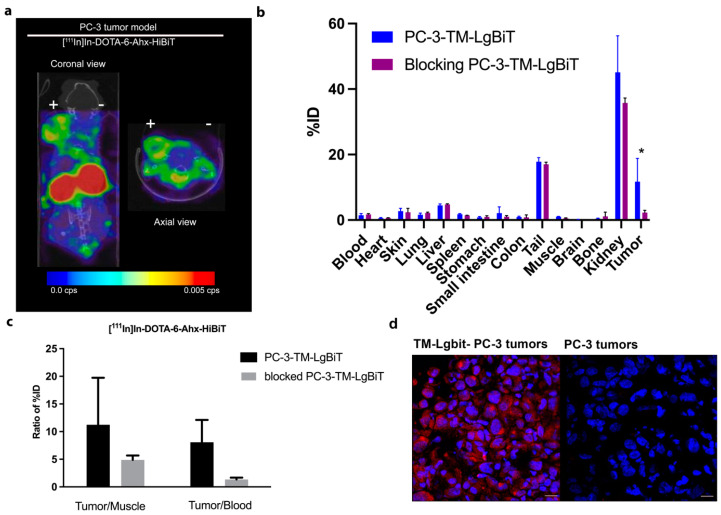
[^111^In]In-DOTA-6-Ahx-VSGWRLFKKIS (Hibit) SPECT/CT imaging and ex vivo biodistribution in PC-3 tumor xenografts. [^111^In]In-DOTA-6-Ahx-VSGWRLFKKIS showed higher uptake in the TM-LgBiT-positive tumor (left flank, +) compared with the TM-LgBiT-negative tumor (right flank, −) at 1 h after i.v. administration of [^111^In]In-DOTA-6-Ahx-VSGWRLFKKIS tracer (**a**). Ex vivo biodistribution confirmed the [^111^In]In-DOTA-6-Ahx-VSGWRLFKKIS specificity since a significant difference in tumor uptake (* *p*-value < 0.05) was detected when performing blocking studies (**b**). Prostate cancer (PC-3) was employed to establish a xenograft model with both target-positive (PC-3-TM-LgBiT) and -negative (PC-3) tumors. Tumor-to-muscle and tumor-to-blood ratios demonstrate specific uptake of [^111^In]In-DOTA-6-Ahx-VSGWRLFKKIS in PC-3 TM-LgBiT tumor, with a reduced ratio in mice that were pre-administered with 100-fold excess of cold In-DOTA-6-Ahx-VSGWRLFKKIS (**c**). Tissue IF of PC-3-TM-LgBiT tumor cells and PC-3 tumor cells using an anti-HA antibody confirm the expression of TM-LgBiT in mouse tumors ( microscope magnification 20X) (**d**).

To evaluate the specificity of this imaging method, we employed mouse tumor models. PC-3-TM-LgBiT cells were injected in BALB/c nude mice (n = 8). The tumors were allowed to grow until they reached a palpable size of approximately ~4–6 mm in diameter. The mice were subjected to a 1 h dynamic SPECT/CT scan after i.v. administration of 0.13 nmol/20 MBq of [^111^In]In-DOTA-6-Ahx-VSGWRLFKKIS. As can be seen in Figure 5a, the signal from the target-positive tumor (PC-3-TM-LgBiT) is delineated, indicating binding of [^111^In]In-DOTA-6-Ahx-VSGWRLFKKIS to the PC-3-TM-LgBiT xenograft. This was confirmed by an ex vivo biodistribution study (Figure 5b), where the uptake of PC-3-TM-LgBiT tumors was shown to be 11.73% ID/g. Non-specific background activity, predominantly in the kidneys (59.3% ID/g), was seen in all animals (Figure 5b). In addition, tumor uptake was blocked by excess of unlabeled peptide, further confirming [^111^In]In-DOTA-6-Ahx-VSGWRLFKKIS specific inhibition. The co-injection of 13 nmol of DOTA-6-AHX-VSGWRLFKKIS (100-fold excess) led to decreased tumor uptake (total of 2.30% ID/g). The tumor-to-muscle uptake ratio for [^111^In]In-DOTA-6-Ahx-VSGWRLFKKIS without blocking was 11.2 ± 0.9, while it was 4.8 ± 0.05 in the blocking studies. The tumor-to-blood ratios were 8.0 ± 3.0 in mice and 1.3 ± 0.3 in the blocking study mouse cohort. (Figure 5c). To confirm the presence of TM-LgBiT in tumors, the cryosections were analyzed with rabbit anti-HA monoclonal antibody for the presence of the chimeric TM-LgBiT reporter construct. Unlike control tumor sections (PC-3), PC-3-TM-LgBiT tumors displayed a clearly visible signal (Figure 5d).

**Figure 6:**
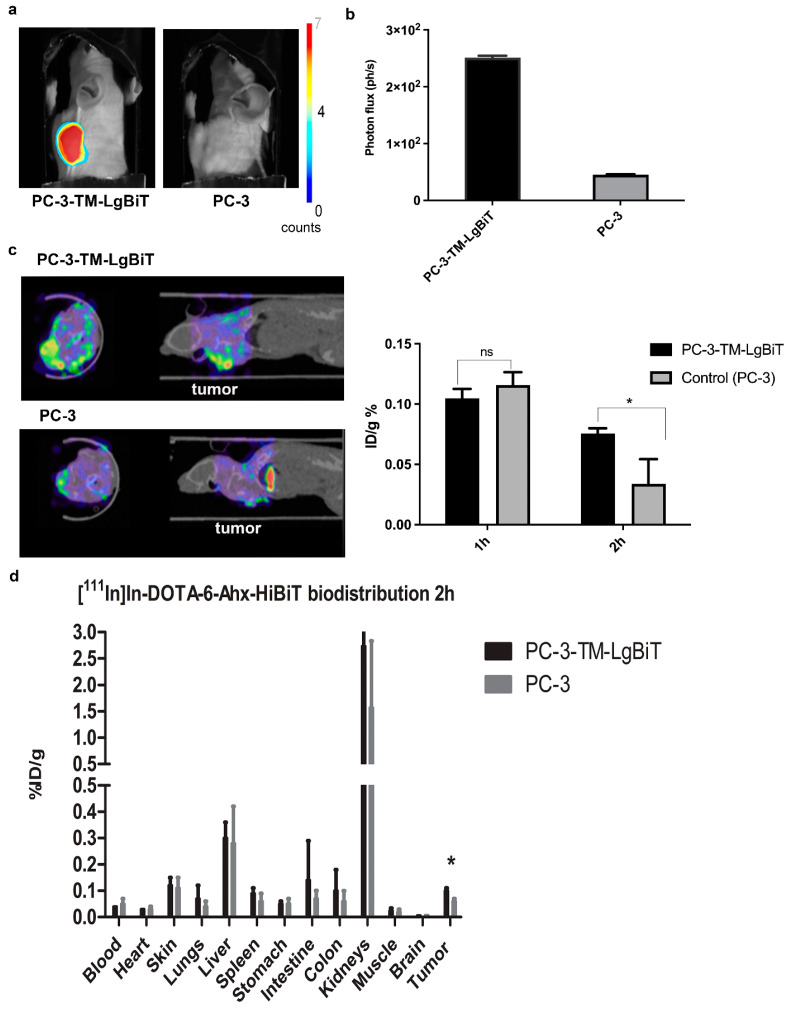
Multimodality imaging of LgBiT-TM-expressing cells. Prostate cancer (PC-3) was employed to establish a xenograft model with both target-positive (PC-3-TM-LgBiT) and -negative (PC-3) tumors. Mice receiving 1 nMol/20 mBq of [^111^In]In-DOTA-6-Ahx-VSGWRLFKKIS were imaged for bioluminescence 15 min after addition of fluorofurimazine substrate (**a**). Tumor-to-background ratio of bioluminescence signal in target-positive and -negative PC3-tumor-bearing mice after administration of fluorofurimazine substrate (**b**). SPECT images of target-positive (PC-3-TM-LgBiT) (**left**) and -negative (PC-3) (**right**) tumors at 2 h after injection of [^111^In]In-DOTA-6-Ahx-VSGWRLFKKIS. Graph reporting the tumor %ID/g calculated from images of tumors at 1 h and 2 h after injection (**c**). Ex vivo distribution study at 2 h after administration of [^111^In]In-DOTA-6-Ahx-VSGWRLFKKIS revealed significant differences between target-positive (PC-3-TM-LgBiT) and -negative (PC-3) tumor uptake (* *p*-value < 0.05; ns = not significantly different) (**d**).

As a final proof-of-concept experiment, we investigated the feasibility of simultaneous BL and SPECT/CT imaging. In addition, we quantified SPECT signals at two imaging time points (1 and 2 h) and determined ex vivo biodistribution at 2 h as further evidence of specificity. Signal specificity was higher (~10-fold) when bioluminescence imaging was employed (Figure 6a,b).

As shown in Figure 6c,d, mice carrying PC-3-TM-LgBiT cells had significantly higher average radioactive signals (~2-fold) in tumors compared to PC-3 controls at the 2 h time point. This was confirmed by the ex vivo biodistribution data at 2 h (Figure 6d).

## 3. Discussion

In this study, we described the development of a chimeric reporter gene based on the NanoLuc binary technology for stable transmembrane (TM) expression of the large NanoLuc subunit (TM-LgBiT), which, when combined with the radiolabeled small NanoLuc subunit (HiBiT probe) [^111^In]In-DOTA-6-Ahx-VSGWRLFKKIS, showed utility for multimodal imaging. We validated the system across critical aspects, i.e., compound synthesis and radiolabeling, in in vitro and in vivo biodistribution studies and BLI and SPECT/CT imaging of implanted HEK-293T-LgBiT cells and PC-3-LgBiT tumors in mice.

In preclinical settings, BLI genes are valuable tools that are relatively inexpensive and allow the tracking of cell migration, cell viability and longitudinal proliferation [24,25]. In clinical applications, the preferred imaging modalities include radionuclide technologies such as SPECT and PET, where the level of measured radioactivity is in direct correlation to the amount of trapped tracer in the desired tissue [26,27]. Unlike visible light, the radiation signals permeate through tissues, enabling sensitive detection in deep tissue (>1 cm) [28]. Our approach combines the sensitivity and medium-throughput capabilities of BLI with the deep tissue specificity and the molecular and anatomical information provided by SPECT/CT.

We aimed to engineer two DOTA-HiBiT peptide conjugates (DOTA-VSGWRLFKKIS and DOTA-6-Ahx-VSGWRLFKKIS) to enable SPECT/CT imaging. Peptides conjugated to DOTA directly or through a linker showed slightly improved affinity toward TM-LgBiT in comparison with the native HiBiT peptide. These results proved that conjugation of the peptide to a linker and/or chelator did not impair binding [25].

Considering that the successful interaction of TM-LgBiT and HiBiT in vivo is dependent on the bioavailability of the HiBiT peptide and its affinity at physiological temperature, we initially investigated whether PC-3-TM-LgBiT tumors could be visualized via BLI imaging using HiBiT or DOTA-6-Ahx-VSGWRLFKKIS. The obtained BLI signal was significantly higher than background, enabling unequivocal detection of tumor cells in mice four weeks after tumor cell implantation. This enables the possibility to employ HiBiT as an imaging tracer for monitoring the in vivo distribution of small molecules, drugs or antibodies tagged with LgBiT.

Initial proof-of-concept in vivo SPECT/CT studies with HEK-293T cells expressing TM-gallium-68 LgBiT and administration of [^111^In]In-DOTA-6-Ahx-VSGWRLFKKIS tracer subcutaneously to nude mice revealed significantly higher uptake in target-positive cells, indicating that the specificity of HiBiT/LgBiT interaction was maintained. We then evaluated the same prostate cancer tumor model (stably expressing TM-LgBiT on the cell membrane) using i.v. [^111^In]In-DOTA-6-Ahx-VSGWRLFKKIS tracer administration. This model revealed a slight difference in tracer uptake in favor of target-positive tumors after two hours. Importantly, with the exception of the kidneys and, to a lesser degree, the liver, no other tissue showed any relevant signal at the 2 h time point, indicating that the HiBiT peptide did not accumulate substantially in non-target tissues. This specificity, combined with the extremely low levels of radiation associated with the imaging radionuclides (e.g., indium-111; gallium-68) and the tracer, likely contributes to the absence of observed toxicity for this imaging technology. Other reporter genes for radionuclide imaging, derived from human receptors, have the disadvantage of uptake in tissues where the receptors are expressed (e.g., hNIS reporter shows uptake in the salivary glands, thyroid and stomach) [17]. Blocking studies using a 100-fold excess of non-radioactive indium-labeled peptide showed a decrease in the SPECT signal in tumors and other organs, demonstrating successful tracer blocking. Most likely, the uptake in background organs at 1 h (except for the kidney, where excretion occurs) is due to blood flow.

Although the fraction of the injected dose taken up in our models is relatively low, we believe that further optimization of the [^111^In]In-DOTA-6-Ahx-VSGWRLFKKIS tracer affinity, the administered dose and the timing of imaging will improve the outcome. Ideally, this would allow the translation to clinical applications. In this regard, NanoLuc luciferase is not a human protein; thus, its potential immunogenicity should be evaluated before any clinical translation. However, therapeutic T cells, for example, are usually administered once in patients with previous lymphodepleting conditioning and subsequent immunosuppression, which mitigates immunogenicity-related issues. Moreover, the employment of different radionuclides for PET (e.g., copper-64) could improve radionuclide imaging. The employment of cytotoxic radionuclides (e.g., 177-lutetium) could open the possibilities for radionuclide therapy following the theragnostic approach and facilitate the elimination of tagged cells.

Note although it was not investigated here, we believe it is possible that LgBiT could also be modified and radiolabeled for the purpose of detecting and imaging HiBiT-tagged targets using BLI and radionuclide imaging.

Ultimately, we believe that these novel multimodality imaging tools, along with other new approaches [29,30,31], show the potential for evaluating gene and cell-based therapies [32,33,34]. Further, we anticipate that these tools will be extremely useful in preclinical studies involving the investigation of cancer progression/regression, metastatic burden and treatment strategies.

## 4. Materials and Methods

### 4.1. Synthesis of HiBiT Peptide

HiBiT (VSGWRLFKKIS) was synthesized using a *Nα*-Fmoc solid-phase peptide synthesis strategy. The conjugation of Fmoc-protected sequence (Val-Ser(*t*Bu)-Gly-Trp(Boc)-Arg(Pbf)-Leu-Phe-Lys(Boc)-Lys(Boc)-Ile-Ser(*t*Bu)) to the 2-chlorotrityl chloride resin was carried out in dimethylformamide (DMF) using hexafluorophosphate azabenzotriazole tetramethyl uronium (HATU) (3.8 equivalent) and *N*,*N*-diisopropylethylamine (DIPEA) (7.8 equivalent) for 45 min. Fmoc deprotection was accomplished via treatment of the resin with a 20% solution of piperidine in DMF. Amide formation and Fmoc deprotection were monitored using the Kaiser test. Double couplings or Fmoc deprotection were performed when the reaction was not completed. The peptide synthesis was started by loading Fmoc-L-Ser(*t*Bu)-OH (1.6 mmol, 4 equivalent) onto the solid support (0.25 g, loading capacity: 1.6 mmol/g). The resin was shaken for 90 min at room temperature. The resin was capped using dichloromethane/methanol/*N*,*N*-diisopropylethylamine (DCM/MeOH/DIPEA) (10 mL, 80:15:5, *v*/*v*/*v*) for 15 min at room temperature. Subsequent Fmoc deprotection and coupling with Fmoc-L-Ile-OH, Fmoc-L-Lys(Boc)-OH, Fmoc-L-Lys(Boc)-OH, Fmoc-L-Phe-OH, Fmoc-L-Leu-OH, Fmoc-L-Arg(Pbf)-OH, Fmoc-L-Trp(Boc)-OH, Fmoc-Gly-OH, Fmoc-L-Ser(*t*Bu)-OH and Fmoc-L-Val-OH were achieved with 4 equivalents of the respective protected amino acids following the protocol described above.

### 4.2. Synthesis of DOTA-6-Ahx-VSGWRLFKKIS Conjugate

The conjugation of the linker to the N-terminal valine residue was accomplished by using Fmoc-6-Ahx-OH (2 equivalent), HATU (3.8 equivalent) and DIPEA (7.8 equivalent) in DMF. The resin was stirred for 2 h at room temperature. Then, the resin was washed thrice with DMF, and the Fmoc protecting group was removed via treatment of the resin with a 20% solution of piperidine in DMF. DOTA-tris(*t*Bu) ester (3 equivalent) was coupled to the peptide in the presence of benzotriazol-1-yl-oxytripyrrolidinophosphonium hexafluorophosphate (PyBOP) (3 equivalent), DIPEA (6 equivalent) and DMF. The reaction was carried out overnight at room temperature. Cleavage of the peptide from the solid support and its concomitant global deprotection were performed via treatment of the resin with a solution of trifluoroacetic acid/water/triisopropylsilane (TFA/H_2_O/TIPS) (95:2.5:2.5, *v*/*v*/*v*) for 6 h at room temperature. Upon filtration, the filtrate was collected, and the solvent was evaporated under vacuum. Trituration of the residue with cold diethyl ether rendered the final crude product, which was purified by HPLC to yield pure DOTA-6-Ahx-VSGWRLFKKIS as a white solid (12 mg, 16.8%). ESI-MS: *m*/*z*, calculated: 1820.40 [M]; found: 910.80 [M + 2H]^2+^, 922.21 [M + Na + H]^2+^.

### 4.3. Preparation of [^111^In]In-DOTA-6-Ahx-VSGWRLFKKIS Peptide

[^111^In]InCl_3_ (93.3 μL, 150 MBq) was added to a mixture of DOTA-6-Ahx-VSGWRLFKKIS (1 nmol), ascorbic acid/gentisic acid (10 μL, 50 mM), sodium acetate (1 μL, 2.5 M) and H_2_O (29.7 μL). The mixture was incubated for 20 min at 90 °C. The reaction was monitored by instant thin-layer chromatography (iTLC) on silica-gel-impregnated glass fiber sheets eluted with a solution of sodium citrate (0.1 M, pH 5.0). The DOTA-chelated indium-111 migrated to the top of the sheet, while the unreacted indium-111 remained on the baseline. The reaction mixture was cooled down for 5 min, and diethylenetriaminepentaacetic acid (DTPA) (5 μL) was added to complex the remaining free indium-111. The radiochemical yield and molar activity of [^111^In]In-DOTA-6-Ahx-VSGWRLFKKIS were determined to be 93% and 150 MBq/nmol, respectively.

### 4.4. Determination of the Distribution Coefficient (LogD_7.4_)

The distribution coefficient (LogD_7.4_) for the ^111^In-labeled compound was determined using the shake-flask method. The experiments were performed in triplicate. A sample containing the radioligand (0.5–2.0 MBq) was dissolved in 1 mL solution of phosphate-buffered saline (0.01 M, pH 7.4) and *n*-octanol (*v*:*v* = 1:1). The vials were vortexed vigorously and then centrifuged at 10,000 RPM for 3 min for phase separation. Samples (10 μL) of each phase were analyzed using a gamma counter. LogD_7.4_ values were calculated using the following equation: LogD_7.4_ = log [(counts in *n*-octanol phase)/(counts in PBS phase)].

### 4.5. Stability Studies in PBS Buffer and Mouse Serum

The stability in PBS was determined by incubating 30 µL of the labeled compounds (~40 MBq) in PBS (70 µL) at 37 °C. Radiochemical purity was determined by iTLC at 30 min, 1 h, 2 h and 4 h. The stability in the serum was carried out by adding 50 µL of the labeled compound (~68 MBq) to 550 µL of mouse serum (Merck, Haarlerbergweg, The Netherlands). The mixture was incubated at 37 °C. At different time points (30 min, 1 h, 2 h and 4 h), an aliquot of the mixture (100 µL) was added to 100 µL of acetonitrile. The vial was vortexed and centrifuged at 10,000 RPM for 20 min, and the supernatant was analyzed by iTLC.

### 4.6. Cell Lines and Cell Culture Conditions

Human embryonic kidney 293T cells (HEK-293T) and human prostate cancer cell line (PC-3) were used for the purposes of this study. Human embryonic kidney (HEK-293T) cells were grown in Dulbecco’s Modified Eagle’s Medium (DMEM) (Sigma-Aldrich, St. Louis, MO, USA), and the PC-3 cell line was cultured in Roswell Park Memorial Institute (RPMI) 1640 Medium (Sigma-Aldrich). Both cell lines were supplemented with 10% fetal bovine serum and 1% penicillin/streptomycin. Cells were cultured in T-175 flasks (Thermo Fisher Scientific, Waltham, MA, USA) until reaching confluence.

### 4.7. Construction of Chimeric Transmembrane LgBiT Reporter (TM-LgBiT), Lentivirus Production and Cell Transduction

Vector production and cell transduction were performed under appropriate biosafety level conditions (ML-II) in accordance with the National Biosafety Guidelines and Regulations for Research on Genetically Modified Organisms (GMO permit 99–163 from the Bureau of genetically modified organisms, The Netherlands). The procedures and protocols were reviewed and approved by the EMC Biosafety Committee. The transmembrane LgBiT sequence (TM-LgBiT) was created via construction of the pCDH1-TmLgBiT-EF1-copGFP lentiviral vector. Initially, we cut the pDisplay vector (Thermo Fisher Scientific), a mammalian expression vector that enables the display of proteins on the cell surface, with restriction enzymes Bgl II and Sal I in NEB 3.1 from New England BioLabs, Ipswich, MA, USA. The LgBiT gene was amplified from the vector pBIT1.3.-C[LgBiT] (Promega, Madison, WI, USA) using the following primers: forward primer with a forward Sal I restriction site 5′-GTCGACGCTGTTGATGGTTAC TCGGAAC-3′ and 5′-AGATCTGTCTTCACACTCGAAGATTTC G-3′ and reverse primer with a Bgl II restriction site. The amplified PCR product (LgBiT gene) was cloned in the above-prepared pDisplay recipient vector (Thermo Fisher Scientific) to create the pDisplay-TM-LgBiT vector using Sal I and Bgl II restriction sites. LgBiT was cloned in a frame with the leader sequence and the C-terminus of the PDGFR transmembrane domain, which anchors the LgBiT protein to the plasma membrane, displaying it on the extracellular side. Recombinant proteins expressed from the pDisplay™ vector contain the hemagglutinin A and myc epitopes. Furthermore, in order to enable cell sorting and stable expression of TM-LgBiT in the cells of interest, we cut the pCDH1-MCS2-EF1-copGFP vector (Addgene, Cambridge, MA, USA), a mammalian expression vector, with restriction enzymes BamHI and NotI in NEB 3.1 buffer from New England BioLabs. The LgBiT sequence was cut from the above-created vector construct (pDisplay-TM-LgBiT) with BamHI and NotI restriction enzymes (New England Biolabs) and inserted into the cloning site of the cut pCDH1-MCS2-EF1-copGFP vector (Addgene, Cambridge, MA, USA) to create pCDH1-TmLgBiT-EF1-copGFP.

To create the pCDH-EF1- LgBiT-T2A-copGFP lentiviral vector, we first excised the 1929 luciferase gene from the vector pCDH-EF1-ATG-1929-T2A-copGFP (Addgene 189713) with restriction enzymes BamHI and NotI in NEB buffer 3.1. from New England Biolabs. The LgBiT gene was amplified from the vector pBIT1.3.-C[LgBiT] (Promega) using the following primers: forward primer with a NotI restriction site 5′-GGGTTTAAACTTAGCTGTTGATGGTTACTC-3′ and 3′-GGATCCATGCTGGCTCGAGCGGTGG-5′ with a BamHI restriction site. The amplified PCR product (LgBiT gene) was cloned in the above-prepared pCDH-EF1-ATG-T2A-copGFP recipient vector to create the pCDH-EF1-LgBiT-T2A-copGFP vector using the NotI and BamHI restriction sites.

Self-inactivating lentivirus particles of pCDH-EF1-LgBiT-copGFP or pCDH1-TmLgBiT-EF1-copGFP were produced via transient transfection of HEK-293T packaging cells with the lentiviral plasmids and the three packaging plasmids, pCMV-VSVG, pMDLg-RRE and pRSV-REV (Addgene, Cambridge, MA, USA), using PEI transfection reagent 1 mg/mL per µg DNA, as previously described in detail by Mezzanotte et al. [24]. After 48 and 72 h, the lentiviral supernatant was collected and filtered through a 0.45 µm membrane. Viral quantification was performed using a standard antigen capture HIV p24 ELISA (ZeptoMetrix, Buffalo, NY, USA). PC-3 cells were grown in culture dishes until reaching 70% confluence and were infected with the above-described lentiviral stock, with the final result of transmembrane LgBiT expression. Cells were transduced by addition of pseudoviral particles at MOI 1 in the presence of polybrine (hexametride bromide, Sigma-Aldrich) at a final concentration of 8 µg/mL. Stable clones were sorted for GFP expression using FACS (BD-FACS AriaIII, BD Biosciences, Franklin Lakes, NJ, USA). Transgene expression was confirmed by the production of green fluorescent protein signal copGFP, excitation/emission maximum = 475/509 nm.

### 4.8. Functionality Assessment of TM-LgBiT Expression

PC-3 cells stably expressing LgBiT reporter at the membrane (TM-LgBiT) and PC-3 cells stably expressing LgBiT within the cytosol were seeded with an equal seeding density (50,000 cells/well) in a black 96-well plate (Greiner-Bio-One, Frickenhausen, Germany). The HiBiT peptide was added to the wells in dilutions ranging from 100 nM^−1^ pM containing the NanoLuc substrate (furimazine), prepared as suggested by the manufacturer (1:50 dilution in PBS). Bioluminescence signals from the wells were acquired at the GloMax^®^ Discover Luminometer (Promega) with 1 s acquisition time immediately after peptide/substrate solution addition. Measurements were performed at room temperature (18–20 °C). Experiments were performed in triplicate and were repeated three times. All data were plotted using GraphPad Prism version 8.

### 4.9. Binding Affinity of Native HiBiT, DOTA-6-Ahx-VSGWRLFKKIS and DOTA-VSGWRLFKKIS Peptides for TM-LgBiT Reporter

The equilibrium dissociation constant (K_D_) was determined by a protocol, as suggested by the manufacturer (Promega), at the GloMax Discover Luminometer (Promega). An OptiMEM solution with 10% fetal bovine serum (FBS) was prepared, where the LgBiT purified protein contained in the Nano-Glo^®^ HiBiT Lytic Detection System (Promega N3030) was diluted in OptiMEM plus 10% FBS to a final concentration of 2 nM from a starting concentration of 200 nM. Serial dilutions of synthetic peptides (DOTA-6-Ahx-VSGWRLFKKIS, DOTA-VSGWRLFKKIS) and native HiBiT were prepared ranging from 200 nM to 2 nM in OptiMEM plus 10% FBS. A volume of 90 µL of the above-prepared solutions was added in triplicate to a white assay plate (Costar 3600), and 10 µL of 2 nM LgBiT solution (0.2 nM final concentration) was added to the wells with the peptide solutions and was incubated in an orbital shaker for 30 min at 600 RPM. A solution of furimazine (Promega) and 1 mM DTT (Thermo Fisher Scientific) was prepared in OptiMEM plus 10% FBS, and 10 µL was added to each well containing the peptide solutions and purified LgBiT. The plate was incubated in an orbital shaker at 600 RPM for 5 min after addition of the solution. Luminescence was measured at the GloMax Discover Luminometer (Promega) with 0.5 s integration time per well. K_D_ was calculated using GraphPad Prism one site specific binding.

### 4.10. Cell-Based Bioluminescence Assay

Both target-expressing (PC-3-TM-LgBiT) and target-non-expressing (PC-3) cells were seeded in a black 96-well plate (Greiner-Bio-One, Frickenhausen, Germany), typically with 10 000 cells per well. The next day, the medium from the wells was removed. DOTA-6-Ahx-VSGWRLFKKIS or the original HiBiT peptide (10 nM and 1 nM) were added to the wells at a final volume of 100 µL. The cells were incubated for 2 h at 37 °C. After the incubation step, peptides were removed and the cells were washed three times with phosphate-buffered saline (PBS) (Lonza, Basel, Switzerland), and the medium was replaced with 50 microliters of OptiMEM. The NanoLuc substrate furimazine from the Nano-Glo Luciferase assay system (Promega) was dissolved in Nanoglo assay buffer and added to all wells, both washed and unwashed. Luminescence images were acquired at the GloMax^®^ Microplate Reader (Promega) with 1 s of integration time. Measurements were performed at room temperature. Experiments were performed in triplicate and were repeated twice. All data were plotted using GraphPad Prism.

### 4.11. Radioactivity Cell Uptake Assay

The membrane-bound fraction of [^111^In]In-DOTA-6-Ahx-VSGWRLFKKIS radiotracer was determined after incubation with 3 different concentrations of [^111^In]In-DOTA-6-Ahx-VSGWRLFKKIS with HEK-293T-TM-LgBiT cells and control cells not expressing TM-LgBiT (HEK-293T). Twenty-four hours before the uptake experiment, cells were seeded in 24-well plates (100,000 cells/well) (Greiner-Bio-One, Frickenhausen, Germany). The next day, cells were incubated with 1 × 10^−8^ M, 1 × 10^−9^ M and 1 × 10^−10^ M of [^111^In]In-DOTA-6-Ahx-VSGWRLFKKIS in 1 mL of culture medium for 1 h at 37 °C. After the incubation step, cells were placed on ice; the supernatant was removed; and cells were washed three times with ice-cold phosphate-buffered saline (PBS) (Lonza). In order to determine the membrane-bound fraction of the peptide tracer ([^111^In]In-DOTA-6-Ahx-VSGWRLFKKIS), cells were incubated for 10 min with an acid solution (50 mM glycine and 100 mM NaCl, pH 2.8). The membrane-bound fraction of the radiotracer was counted in a γ-counter (1480 WIZARD automatic g counter; PerkinElmer, Waltham, MA, USA) using a radionuclide-specific energy window, a counting time of 60 s and a counting error of 5% or less. Data are expressed as a percentage of added dose.

### 4.12. Bioluminescence Imaging

BALB/c nude (males) were obtained from Charles River Laboratory (‘s-Hertogenbosch, The Netherlands). All mice aged 8 weeks were provided access to food and water ad libitum and were hosted in the animal facility at the Erasmus MC, Rotterdam, The Netherlands.

Prior intravenous peptide administration (HiBiT and DOTA-6-Ahx-VSGWRLFKKIS) background signal was estimated: tumor-bearing mice (PC-3-TM-LgBiT) were intraperitoneally (i.p) injected with the recently formulated water-soluble substrate fluorofurimazine (FFz) [35], with a final concentration of 1.3 µmol in a total volume of 120 µL. Mice were anesthetized with isoflurane (1.5%) and imaged after substrate administration at the IVIS Spectrum (PerkinElmer). Prone BLI images were acquired at the IVIS (exposure time: 30 s). Regarding the bioluminescence light assessment, nude BALB/c mice (n = 6) with subcutaneously implanted PC-3-TM-LgBiT cells stably expressing TM-LgBiT were intravenously injected with 1 nmol of DOTA-6-Ahx-VSGWRLFKKIS or HibiT in a total volume of 100 µL. Thirty minutes after peptide administration, all mice received an intraperitoneal (i.p.) injection of fluorofurimazine FFz (1.3 µmol). Mice were kept under isoflurane anesthesia (1.5%), and a series of images were taken using an IVIS spectrum (PerkinElmer) with open filter; binning = medium, field of view = 12.9 cm × 12.9 cm, f/stop = 1, exposure time = 1 min every 5 min for 20 min. Data analysis was performed by drawing the ROIs in the images taken at the peak of bioluminescence emission using Living Image software 4.2 (Perkin Elmer). Signals were corrected for tumor volume.

### 4.13. SPECT/CT Imaging

For the subcutaneous injection, HEK-293T-LgBiT model, 8–10-week-old BALB/c nude (male) mice (n = 3) were implanted with 1 × 10^6^ HEK-293T-TM-LgBiT cells in the right flank and 1 × 10^6^ HEK-293T (control) in the left flank. Regarding the subcutaneous tumor model, 8-week-old nude BALB/C male mice (n = 12) were injected with 5 × 10^6^ PC-3-TM-LgBiT-expressing cells (n = 8). Both cell lines were prepared for injections in PBS (Sigma-Aldrich) and a matrigel (Corning) solution with a 50:50 ratio and a final injectable volume of 50 µL. Tumors were left to grow approximately 3–4 weeks after tumor cell implantation. To determine the functionality of our imaging system, a dynamic whole-body SPECT/CT scan (VECTOr/CT with additional optical unit Milabs) was performed. Mice were anesthetized using 1–2% isoflurane/O_2_, and the body temperature was maintained at 37 °C during the time of imaging (1 h) by using a heated bed aperture. The 1 h dynamic SPECT/CT scan was performed immediately after tail vein injection of [^111^In]In-DOTA-6-Ahx-VSGWRLFKKIS in the PC-3 tumor model or subcutaneous injection of [^111^In]In-DOTA-6-Ahx-VSGWRLFKKIS in the case of the HEK-293T cell model (20 MBq labeled as 0.13 nmol in 200 µL PBS). In order to determine the effect of the unlabeled peptide on PC-3 tumor uptake, 13 nmol of DOTA-6-Ahx-VSGWRLFKKIS (approximately 100-fold excess) was co-injected along with [^111^In]In-DOTA-6-Ahx-VSGWRLFKKIS (n = 4). Dynamic scans were obtained over a total duration of 1 h with 30 time frames directly after injection of [^111^In]In-DOTA-6-Ahx-VSGWRLFKKIS. In the second experiment, 1 h and 2 h SPECT/CT scans of the tumor area were performed after tail vein injection of 20 MBq labeled as 1 nmol of [^111^In]In-DOTA-6-Ahx-VSGWRLFKKIS in 200 µL of PBS in animals bearing both the PC-3 and PC3-TM-LgBiT tumor model (n = 4). The acquired images were reconstructed using SR-OSEM with 9 iterations and 128 subsets on a 36 mm × 36 mm × 35 mm matrix with 0.80 mm × 0.80 mm isotropic voxels. The images were further analyzed in Pi-Mod and VivoQuant. The regions of interest were manually drawn around the tumors, heart and muscle. Subsequently, the percentage of injected dose (%ID) and the tumor-to-background ratio (TBR) were determined.

### 4.14. Ex Vivo Analysis: Biodistribution

Mice were sacrificed and dissected one hour after tail vein injection of approximately 20 MBq [^111^In] In-DOTA-6-Ahx-VSGWRLFKKIS peptide (approximately 20 MBq, 1 nmol, n = 4 per treated group/control). The organs (blood, heart, skin, lungs, liver, spleen, stomach, small intestine, colon, tail, muscle, brain, tumor, kidney and bone) were weighed, and the radioactivity uptake in tumor and other organs was determined and expressed as a percentage of injected dose per gram of tissue (%ID/g). Tumors and organs were counted in a γ-counter (PerkinElmer). The counting time was 60 s per sample with an isotope-specific energy window and a counting error not exceeding 5%. After counting, the tumors were frozen in liquid nitrogen for further analysis.

### 4.15. Immunofluorescence Staining

For immunofluorescence staining of tumor tissue, the frozen xenografts were embedded in Tissue-Tek O.C.T Compound (Sakura Finetek, Alphen aan den Rijn, The Netherlands) and trimmed via cryostat (10 μm) and transferred to glass slides (Thermo Fisher Scientific). Xenografts were fixed with 4% formalin for 15 min at room temperature. After washing with PBS (Sigma-Aldrich), the sections were blocked with 5% normal serum + 0.3 M glycine (Sigma-Aldrich) in TBS/Tween for 1 h at room temperature. The slides were washed in PBS and further on incubated with primary rabbit monoclonal antibody to HA tag (ab236632; Abcam, Cambridge, UK) at a ratio of 1:300 in TBS/Tween + 1% normal serum in a wet chamber overnight at 4 °C. The next day, after the washing step with PBS, the sections were incubated with the secondary antibody goat anti-rabbit Alexa Fluor 594 nm (ab150080; Abcam) at a ratio of 1:1000 in TBS/Tween + 1% normal serum for 1 h at room temperature. The slides were washed with PBS; the nuclei were stained with Hoechst (Thermo Fisher Scientific) at a ratio of 1:1000 in TBS for 5 min at room temperature. The slides were rinsed in distilled water and mounted with prolong diamond antifade (Thermo Fisher Scientific) and analyzed via fluorescent microscopy (Leica Microsystems, Wetzlar, Germany).

### 4.16. Statistical Analysis

Analysis of the data output was performed using column statistics (t-test). Where more than two groups were compared, one-way ANOVA followed by Tukey’s *t*-test were applied. All statistical analyses were performed using GraphPad Prism version 8 for Windows. Data from each condition or animal group were presented as means and SD. The results were statistically significant when *p* < 0.05.

## 5. Patents

The reporter gene technology described in this paper is protected by patent WO2021194343A1 by LM and Erasmus Medical Center.

## Data Availability

All data are available in the main text or the Supplementary Materials.

## Supplementary Materials

The supporting information can be downloaded at: https://www.mdpi.com/article/10.3390/ijms25158206/s1.
