# Supplementary material for: A Novel Luciferase-Based Reporter Gene Technology for Simultaneous Optical and Radionuclide Imaging of Cells
Source: Int J Mol Sci. 2024 Jul 27;25(15):8206. doi: 10.3390/ijms25158206 (PMC11312113; doi:10.3390/ijms25158206)
Supplement: Supplementary file 1 [file ijms-25-08206-s001.zip › ijms-3081381-supplementary.pdf]

Supplementary Materials for

A novel luciferase based reporter gene technology for simultaneous optical and radionuclide imaging of cells

Authors

Natasa Gaspar, Maryana Handula, Marcus C. M. Stroet, Kranthi Marella-Panth, Joost Haeck, Thomas A. Kirkland, Mary P. Hall, Lance P. Encell, Simone U. Dalm, Marion De Jong, Clemens Lowik, Yann Seimbille, Laura Mezzanotte*

*** Corresponding author:** l.mezzanotte@erasmusmc.nl

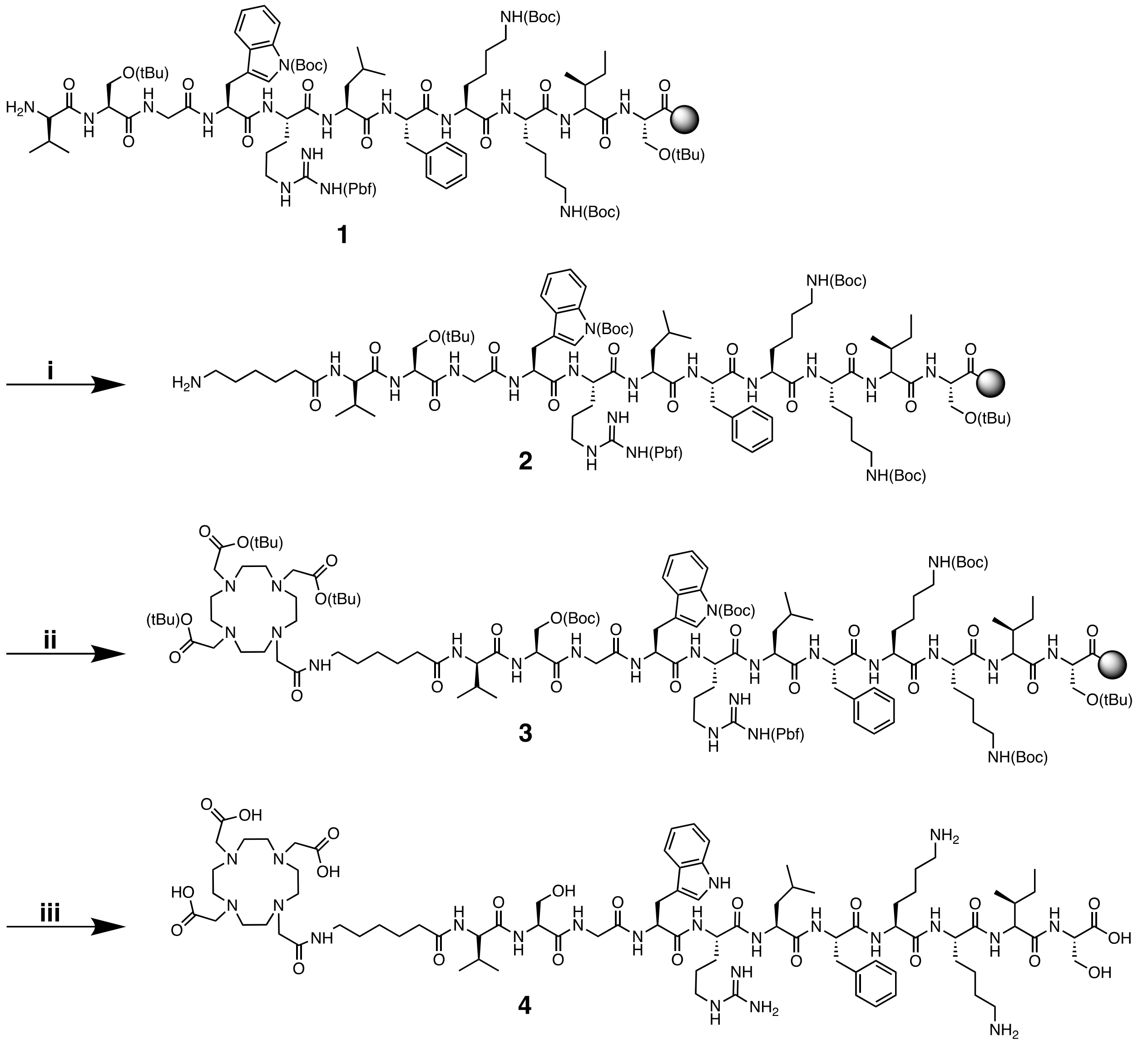

Figure S1. Synthesis of DOTA-6-Ahx-VSGWRLFKKIS. Reagents and conditions: (i) Fmoc-6-Ahx-OH, HATU, DIPEA, 2 h, rt and 20% piperidine in DMF; (ii) DOTA-tris(tBu) ester, PyBOP, DIPEA, o.n., rt; (iii) TFA/H2O/TIS, 6 h, rt.

Supplementary Table S1. Radiochemical yield (RCY), stability in PBS (n = 1) and mouse serum (n = 1), Log$D_{7.4}$ (n = 3) of [^{111}In]In-DOTA-6-Ahx-VSGWRLFKKIS.

**Radioligand**	**RCY (%)**	**Stability in PBS (%)**				**Stability in Mouse Serum (%)**				**Log $D_{7.4}$**
		**30 min**	**1 h**	**2 h**	**4 h**	**30 min**	**1 h**	**2 h**	**4 h**	
[^{111}In]In-DOTA-6-Ahx-VSGWRLFKKIS	96.7	87.4	92.3	90.3	90.1	99.8	89.7	85.2	71.2	-2.0 ± 0.72
